# MELIF, a Fully Automated Liver Function Score Calculated from Gd-EOB-DTPA-Enhanced MR Images: Diagnostic Performance vs. the MELD Score

**DOI:** 10.3390/diagnostics12071750

**Published:** 2022-07-20

**Authors:** Carolina Río Bártulos, Karin Senk, Ragnar Bade, Mona Schumacher, Jan Plath, Nico Kaiser, Isabel Wiesinger, Sylvia Thurn, Christian Stroszczynski, Abdelouahed El Mountassir, Mathis Planert, Jan Woetzel, Philipp Wiggermann

**Affiliations:** 1 Institut für Röntgendiagnostik und Nuklearmedizin, Städtisches Klinikum Braunschweig gGmbH, 38126 Braunschweig, Germany; a.el-mountassir@klinikum-braunschweig.de (A.E.M.); m.planert@klinikum-braunschweig.de (M.P.); p.wiggermann@klinikum-braunschweig.de (P.W.); 2 Institut für Röntgendiagnostik, Universitätsklinikum Regensburg, 93053 Regensburg, Germany; karin.senk@klinik.uni-regensburg.de (K.S.); sylvia.thurn@klinik.uni-regensburg.de (S.T.); christian.stroszczynski@klinik.uni-regensburg.de (C.S.); 3 MeVis Medical Solutions AG, 28359 Bremen, Germany; ragnar.bade@mevis.de (R.B.); mona.schumacher@mevis.de (M.S.); jan.plath@mevis.de (J.P.); nico.kaiser@mevis.de (N.K.); jan.woetzel@mevis.de (J.W.); 4 Institut für Neuroradiologie, Medbo Bezirksklinikum Regensburg, 93053 Regensburg, Germany; isabel.wiesinger@medbo.de

**Keywords:** liver function, T1 relaxometry, MELIF, MELD, MRI, artificial intelligence

## Abstract

In the management of patients with chronic liver disease, the assessment of liver function is essential for treatment planning. Gd-EOB-DTPA-enhanced MRI allows for both the acquisition of anatomical information and regional liver function quantification. The objective of this study was to demonstrate and evaluate the diagnostic performance of two fully automatically generated imaging-based liver function scores that take the whole liver into account. T1 images from the native and hepatobiliary phases and the corresponding T1 maps from 195 patients were analyzed. A novel artificial-intelligence-based software prototype performed image segmentation and registration, calculated the reduction rate of the T1 relaxation time for the whole liver (rrT1_liver_) and used it to calculate a personalized liver function score, then generated a unified score—the MELIF score—by combining the liver function score with a patient-specific factor that included weight, height and liver volume. Both scores correlated strongly with the MELD score, which is used as a reference for global liver function. However, MELIF showed a stronger correlation than the rrT1_liver_ score. This study demonstrated that the fully automated determination of total liver function, regionally resolved, using MR liver imaging is feasible, providing the opportunity to use the MELIF score as a diagnostic marker in future prospective studies.

## 1. Introduction

The determination of liver function (LF) is becoming increasingly important given the rise in chronic liver disease (CLD) and the accompanying increase in the number of patients with liver cirrhosis and hepatocellular carcinoma (HCC) [1,2,3,4,5]. Therefore, a regular assessment of liver function is required for patients with CLD. In addition, the evaluation of liver function prior to liver resection is particularly important for avoiding posthepatectomy liver failure (PHLF) [6]. In clinical practice, blood values are used as a static test to assess liver function [7,8] and are therefore an integral part of scoring systems such as the model for end-stage liver disease (MELD) [9]. The MELD was originally developed as a model to predict survival after transjugular intrahepatic portosystemic shunt (TIPS) [10] and has been expanded for use in the prediction of 3-month mortality in patients with end-stage chronic liver disease awaiting liver transplantation [11,12]. Therefore, its score is often used as a point of reference for the development of new methods to determine liver function.

One promising method for assessing liver function is the use of imaging modalities such as MRI [6,13]. MRI examinations with liver-specific contrast agents such as Gd-EOB-DTPA (gadoxetic acid) allow not only the characterization of liver lesions but also the determination of regional liver function [14]. Evidence from an early study with a small number of patients showed that functional values derived from MR images could predict PHLF in patients undergoing major liver resection [15]. Furthermore, several publications have demonstrated the correlation of the liver function scores determined from MR images with the MELD score [16,17,18]. However, several parameters derived from contrast-enhanced MRI are based on either signal intensity measurements (SI) or T1 relaxometry [14,19]. The latter variant seems to be the method of choice, as the indices derived from the T1 relaxation time show better diagnostic performance [13,20]. The T1 relaxation time is a function of the rate of energy transfer from the excited proton to the surrounding environment and is measured in milliseconds, making it a quantifiable unit, whereas the signal intensity values are not; moreover, the T1 relaxation time is directly related to the contrast agent concentration in hepatocytes [21,22]. In comparative studies, the reduction rate of the T1 relaxation time (rrT1) has been shown to be better correlated with global liver function values than other T1 relaxometry or SI indices [13,20,23], which explains why rrT1 has gained importance as a liver function parameter. Although the studies mentioned here have shown that rrT1 can serve as a good diagnostic parameter, it has thus far only been used in research. One reason for this is the tedious and time-consuming nature of determining this value. It is necessary to manually place the regions of interest (ROIs) on the T1 maps and then insert the T1 relaxation times obtained into the equation to calculate rrT1 [24,25]. Consequently, MRI-based strategies for determining liver function remain in the investigational phase [26]. The reason for this is the lack of suitable software for automatically calculating liver function values such as rrT1 from MR images.

In this study, an artificial intelligence (AI)-based software prototype was used to process MR images, enabling liver segmentation, image registration, and the quantification of liver function in a single step. This software calculates the rrT1 value for the entire liver (rrT1_liver_) fully automatically, taking each voxel into account, not only individual ROIs or selected slices. In addition, the software calculates a liver function value (MELIF) optimized for the patient’s size, weight and liver volume. Thus, the software enables the assessment of regional liver function. The aim of this study was to evaluate fully automatically computed liver function scores (MELIF and rrT1_liver_) based on Gd-EOB-DTPA T1 relaxometry in terms of their diagnostic performance in liver diseases with respect to the commonly used liver function score, the MELD score.

## 2. Materials and Methods

### 2.1. Study Design and Subjects

This retrospective study was approved by the Institutional Review Board, and international and national regulations for handling patient data were followed.

For this retrospective study, the records of 195 consecutive patients who underwent Gd-EOB-DTPA-enhanced MRI were used. Gd-EOB-DTPA was used as a liver-specific contrast agent and administered as a body-weight-adapted bolus injection, as specified by the manufacturer (Primovist©, Bayer Schering Pharma AG, Berlin, Germany). MRI examinations were performed as part of the routine clinical examination of the patients.

Some of the purposes of the scan were to evaluate an unknown liver lesion revealed by other examinations (ultrasound or computed tomography) to exclude metastases, search for metastases in malignant primary disease, follow up after the treatment of a focal malignant liver lesion, and control the progression of CLD or known lesions. None of the patients had a contraindication for MRI examination or contrast agent administration, and all patients consented in writing to the examination.

The MELD was used as an established clinical scoring system to assess liver function, and its score was calculated according to the formula published in [10,17]. The laboratory parameters used to calculate the MELD score were obtained no more than 24 h before or after the MRI examination. Consequently, patients were divided into three groups based on the MELD score. A MELD score of 10 or less was considered to indicate normal liver function; between 11 and 18, impaired liver function; and above 18, severely impaired liver function [12,17].

### 2.2. Image Acquisition and Analysis

MR imaging was performed on a 1.5T system (Magnetom Symphony, Siemens, Erlangen, Germany) for 45 data sets and on a 3T system (Magnetom Skyra, Siemens Healthineers, Erlangen, Germany) in combination with an appropriate body array coil for the remaining 150. More details on the MRI sequences for T1 mapping on the 1.5T system are described in Haimerl et al., 2014 [27], and for the 3T system, in Haimerl et al., 2015 [25]. Here, T1 maps based on the variable flip angle (VFA) technique with B1 inhomogeneity correction were obtained before and after contrast agent administration. These T1 maps, as well as hepatobiliary phase (HBP) T1 images, were used for a software prototype that performs automatic liver segmentation and registration and automatically quantifies liver function.

**Figure 1:**
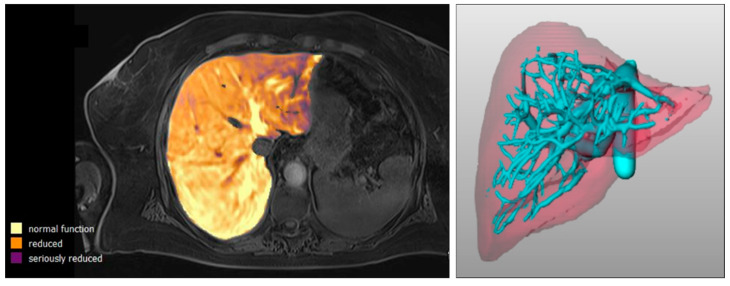
Example of image analysis. Visualization of liver function over the entire liver with the AI-based software prototype (**left**) and 3D view of the liver and vascular supply (**right**).

The quantification of liver function is based on the reduction rate of the T1 relaxation time (rrT1, Equation (1)) [24,25]:

(1) $$\mathrm{rrT}1=\left(\frac{T1_{\mathrm{pre}}-T1_{\mathrm{post}}}{T1_{\mathrm{pre}}}\right)\times 100\;\left(\%\right)$$

where T1_pre_ and T1_post_ represent, in other studies, the T1 times of manually placed regions of interest (ROIs) on T1 maps before and after contrast administration and therefore represent only a part of the liver. Using the prototype software mentioned above, which uses an AI model for liver segmentation, rrT1 can be determined for the entire liver. In addition, the automatic elastic registration of T1_post_ onto T1_pre_ achieves the required spatial correspondence of the exact reduction rate at the voxel level. Thus, regional liver function is provided over the entire liver (see Figure 1).

By calculating the sum of rrT1 over all voxels of the liver, a complete liver-related reduction rate (rrT1_liver_, Equation (2)) is obtained.

(2) $$\mathrm{rrT}1_{\mathrm{liver}}=100\left(\%\right)\times \overset{\mathrm{liver}}{\underset{x,y,z}{\sum }}\frac{T1_{\mathrm{pre}}\left(x,y,z\right)-T1_{\mathrm{postReg}}\left(x,y,z\right)}{T1_{\mathrm{pre}}\left(x,y,z\right)}$$

However, various studies have shown that the rrT1 value correlated with liver volume is a better diagnostic marker [25,28]. Given that the rrT1_liver_ value is summed over all liver voxels, it naturally includes liver volume. Liver volume may vary from one patient to another and also depends on age and severity of disease. Therefore, in liver transplants from living liver donors, for example, the patient’s height and weight are also considered to avoid a graft that is too small [29,30,31]. To take this into account, a patient-specific liver function factor *f_p_* (Equation (3)) was included in the calculation of the liver function value.

(3) $$f_{p}=\frac{{\mathrm{height}}_{p}^{0.6}}{{\mathrm{weight}}_{p}^{0.3}\times {\mathrm{liver}\mathrm{volume}}^{0.6}}$$

This factor includes not only liver volume but also patient height and weight. These additional parameters and their exponents can be determined using linear regression models to model the MELD score. Furthermore, the software uses a constant (*c* = 0.694) to scale the resulting score to a convenient range of values from 20 to 80. The combination of the total liver reduction rate (rrT1_liver_), the patient-specific factor (*f_p_*), and the constant (*c*) yields the formula for optimized liver function quantification (MELIF score; Equation (4)).

(4) $$\mathrm{MELIF}=c\times f_{p}\times \mathrm{rrT}1_{\mathrm{liver}}$$

### 2.3. Statistical Analysis

All statistical analyses were performed with the program GraphPad Prism 9.1.2 (GraphPad Software, LLC, San Diego, CA, USA). Categorical variables are presented as absolute values and percentages, and continuous variables are presented as the median (interquartile range (IQR)) or mean (±standard deviation). Data were tested for normal distribution using the D’Agostino and Pearson test in order to determine the most appropriate method of analysis (parametric vs. nonparametric). The data were normally distributed, so parametric analyses were performed. Only the >18 MELD group was both not “normally” and not “nonnormally” distributed, so parametric analyses were also performed here for comparison. This problem resulted from the small amount of data in this group. Data were compared using descriptive statistics and Pearson’s correlations, including simple linear regression. Paired differences were calculated using the unpaired t test. The area under the receiver operating characteristic curve (AUC) was used for a direct comparison of the two scores. In all analyses, a two-tailed *p* value < 0.05 was considered statistically significant. The correlation coefficient was interpreted as follows: up to 0.1 as weak, 0.3 as moderate and above 0.5 as strong. The AUCs were interpreted as follows: 0.9–1 = excellent, 0.8–0.9 = good, 0.7–0.8 = fair, 0.6–0.7 = poor and 0.5–0.6 = failed.

## 3. Results

A total of 195 data sets from two MRI scanners (1.5 T and 3 T) were used for the study. In 30% (N = 59) of these patients, partial liver resection had been performed previously; one patient had received a liver transplant; and five patients had received a transjugular intrahepatic portosystemic shunt (TIPS). The average patient age was 62 (± 11) years, and 79% were male (Table 1). The mean MELD score for all patients was 9 (IQR = 7–11). On average, the MELIF value of all patients was 51 (±13), and the rrT1_liver_ value was 50 (±12)%. The patient demographic and clinical characteristics are summarized in Table 1.

Using the MELD score as the reference, patients were divided into three groups to reflect the severity of disease. A MELD score of ≤10 indicates normal liver function, scores of 11 to 18 indicate impaired liver function, and scores above 18 indicate severely impaired liver function [12,17]. As shown in Table 2, 68% (N = 132) of the patients belonged to the MELD ≤ 10 group, 30% (N = 59) to the group with impaired liver function, and 2% (N = 4) to the group with severely impaired liver function. The mean age was comparable in the three groups, and the distribution of male and female patients was similar.

**Figure 2:**
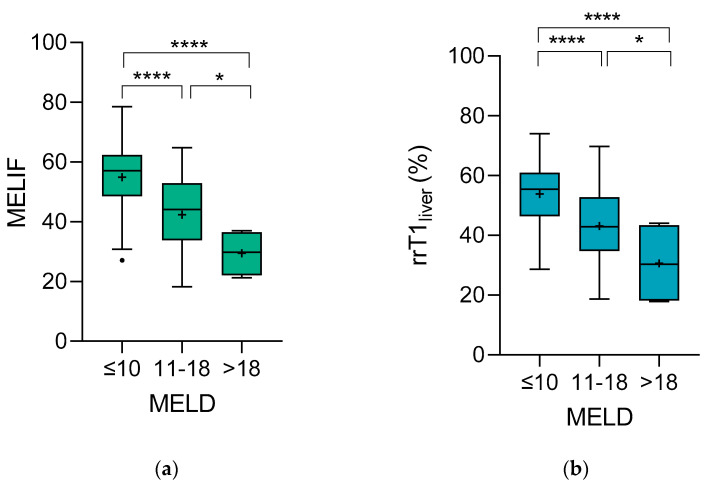
Pairwise comparisons of the MELD groups for (**a**) the MELIF score and (**b**) rrT1_liver_. The boxplots were made according to Tukey’s test. The + represents the mean value. Using the unpaired *t* test, pairwise differences were calculated. **** ≤ 0.0001; * ≤ 0.05. MELD ≤ 10, N = 132; MELD 11–18, N = 59; MELD > 18, N = 4.

The mean MELIF score of the MELD ≤ 10 group was 55 (±11), that of the MELD 11–18 group was 42 (±11) and that of the MELD > 18 group was 29 (±7.7) (Table 2). A pairwise comparison of the mean values showed that they were statistically significantly different (Figure 2a). A very similar finding was observed for the rrT1_liver_ value. The mean rrT1_liver_ score was 54 (±10)% for the MELD ≤ 10 group, 43 (±12)% for the MELD 11–18 group, and 31 (±14)% for the MELD > 18 group (Table 2). In this case as well, pairwise comparisons of the groups showed that the mean values were significantly different (Figure 2b).

**Figure 3:**
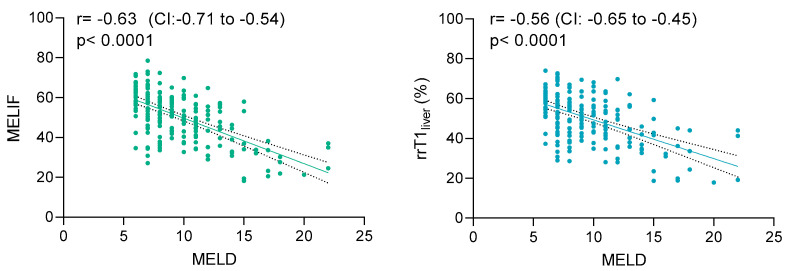
Pearson’s correlations (r) of the MELIF score (**left**) and rrT1_liver_ (**right**) with the MELD score. The correlation coefficient with the 95% confidence interval (CI) is given in the upper left corner of each graph. Simple linear regression was used (straight line), and the CI is shown as a dotted line.

To analyze the relationship between the MELIF and rrT1_liver_ scores and the MELD score, Pearson’s correlation was used (Figure 3). The MELIF score showed a significantly strong negative correlation with the MELD score (r = −0.63). However, this correlation was stronger than the correlation between the rrT1_liver_ score and the MELD score. Nevertheless, the rrT1_liver_ score also showed a significantly strong negative correlation with the MELD score (r = −0.56).

An analysis of the AUC was used to directly compare the two scores (Table 3). The MELIF score showed a good performance (0.79), while the rrT1_liver_ score showed a fair performance (0.75).

## 4. Discussion

MRI seems to be the most promising modality in terms of its diagnostic value for liver anatomy and liver function. It allows the determination of important anatomic information, such as lesion volume; vascular supply; and, using contrast agents such as Gd-EOB-DTPA, regional liver function [14]. However, given the global nature of the methods currently in clinical use, the regional assessment of liver function remains the main argument in favor of these techniques. The regional determination of liver function is important, e.g., for planning surgical procedures, as liver function may be unevenly distributed, as known from scintigraphic procedures [33]; furthermore, there is evidence of an inhomogeneous distribution of liver function in liver cirrhosis [34]. However, as mentioned in the introduction, rrT1 is gaining increasing importance as a factor for determining regional liver function.

In this study, a software prototype was used that determines the rrT1 value fully automatically. This eliminates the need for time-consuming ROI generation and manual calculation for this regional liver function value, providing an objective value without bias. In addition, the image registration of native and contrast-enhanced MR images provides the spatial correspondence required for the accurate determination of rrT1 at the voxel level. Therefore, this allows the rrT1 value to be determined for the whole liver (rrT1_liver_) fully automatically. The mean value for rrT1_liver_ determined in this study is slightly lower than the results from other publications [17,35]. However, the rrT1_liver_ score also showed a strong correlation with the MELD score, confirming other results [35], thus demonstrating the reliability and comparability of the automatically determined rrT1_liver_ value. Furthermore, the mean values differed statistically between the group with normal liver function (MELD ≤ 10) and that with impaired liver function (MELD 11–18, MELD > 18), highlighting the diagnostic significance of this potential biomarker.

Early in the research of liver function values derived from MR images, the question of whether the inclusion of liver volume would provide a better functional value was posed. Liver function values determined by SI-based and volume-normalized methods have been shown to be better correlated with global liver function values [13,36,37,38,39]; likewise, this has been shown for rrT1 [25]. Furthermore, for rrT1, Duan et al. demonstrated that a volume-based rrT1 value performed better in discriminating between patients with normal and abnormal liver function [28]. By calculating rrT1 in a voxelwise manner, the liver volume is included in rrT1_liver_. However, liver volume changes with age and disease; therefore, height and weight factors are considered in liver transplantation [29]. Therefore, the MELIF score takes this concept into account; similar to the rrT1_liver_ score, the MELIF score is also able to significantly discriminate between the MELD groups. However, compared with the rrT1_liver_ value, it shows a stronger negative correlation with the MELD score, thus showing that here, too, the introduction of patient-specific factors leads to a higher correlation. Furthermore, AUC analysis showed that the MELIF score was a more accurate parameter than the rrT1_liver_. This introduces a novel method for determining liver function from MRI data that is fully automated, based on rrT1, and represents a patient-specific and regional liver function score—the MELIF score.

The present study has certain limitations. First, it is based on retrospective data from a single center. However, we assume that this is not a serious limitation, as this is a conformity study with a focus on testing fully automatically generated scores for their diagnostic performance, and in this respect, a large number of patients as well as images from different scanners are more relevant. In addition, we only used the MELD score as a reference value and did not include any other global liver function scores. However, the MELD score has already been confirmed as a reference value in many studies, so this is negligible in this case. Furthermore, underlying diseases were not the focus of this study and consequently were not addressed. Narrowing the score to a patient population or to specific diseases would add value in terms of prognostic significance. Additionally, future studies need to show how useful and applicable the scores presented could be in clinical practice, ideally in prospective trials.

## 5. Conclusions

To bring liver function determination from MR images out of its infancy and into clinical practice, software is required to perform all necessary image analysis tasks, preferably in a fully automatic manner: the segmentation of the liver to obtain a correct liver volume, the precise image registration of native and contrast-enhanced scans, and the fully automated determination of a function score. The software prototype used here was developed precisely for this purpose. Such software provides a basis for determining regional or segmental liver function in the future.

Contrast-enhanced MRI of the liver is routinely used in the evaluation of liver lesions. It provides accurate anatomic information and visualizes liver lesions, making it an indispensable part of preoperative assessment. Therefore, a liver function score that can be determined noninvasively and directly from the MRI examination offers great potential for seamless integration into the clinical examination workflow. The diagnostic potential of such MRI-based scores has been demonstrated in many, mostly retrospective, studies, as mentioned in the introduction. Here, we tested two fully automatically generated MR image-derived regional liver function scores for their diagnostic performance and showed that the personalized liver function score (MELIF), which incorporates patient-specific factors, correlates better with the MELD score than the whole liver rrT1 score.

## Figures and Tables

**Table 1:** Demographic and clinical characteristics of the subjects.

	All (N = 195)
Sex (M/W)	155/40 (79%/21%)
Age (years)	62 (±11)
Height (m)	1.7 (±0.08)
Weight (kg)	83 (±16)
Liver volume (mL)	1513 (±415)
MELD score	9 (7–11)
MELIF	51 (±13)
rrT1_liver_ (%)	50 (±12)

Data are presented as N (%) for categorical variables and mean (±standard deviation) or median (interquartile range) for continuous variables. MELD = model for end-stage liver disease.

**Table 2:** MELIF score, rrT1_liver_, and demographic characteristics by MELD group.

	MELD ≤ 10 (N = 132)	MELD 11–18 (N = 59)	MELD > 18 (N = 4)
Sex (M/W)	104/28 (79%/21%)	48/11 (81%/19%)	3/1 (75%/25%)
Age (years)	61 (±12)	64 (±9.1)	64 (±5.3)
MELIF	55 (±11)	42 (±11)	29 (±7.7)
rrT1_liver_ (%)	54 (±10)	43 (±12)	31 (±14)

Data are presented as N (%) for categorical variables and the mean (±standard deviation) for continuous variables. MELD = model for end-stage liver disease.

**Table 3:** AUC analysis of the MELIF score and rrT1_liver_ score with corresponding sensitivity and specificity values to distinguish patients with normal (MELD ≤ 10) and impaired liver function (MELD 11–18).

	AUC (95% CI)	*p*	Cut Off ^†^	Sensitivity (%)	Specificity (%)
MELIF	0.790 (0.72 to 0.86)	<0.0001	56.78	93.22	51.52
rrT1_liver_	0.755 (0.68 to 0.83)	<0.0001	46.93	66.1	75

CI = confidence interval; ^†^ = calculated using the Youden index [32].

## Data Availability

All relevant data are within the manuscript. Due to the vulnerable nature of the patient data, the anonymized data used for the study are not stored in a publicly accessible database. However, they are available from the authors upon request.
